# Harm reduction workforce, behavioral health, and service delivery in the USA: a cross-sectional study

**DOI:** 10.1186/s12954-024-00952-9

**Published:** 2024-02-10

**Authors:** Lisa de Saxe Zerden, Orrin D. Ware, Brooke N. Lombardi, Brianna M. Lombardi

**Affiliations:** 1 School of Social Work, University of North Carolina at Chapel Hill, 325 Pittsboro St. CB #3550, Chapel Hill, NC 27599 USA; 2 Cecil G. Sheps Center for Health Services Research, University of North Carolina at Chapel Hill, Chapel Hill, USA; 3 Department of Family Medicine, University of North Carolina at Chapel Hill, Chapel Hill, USA

**Keywords:** Harm reduction, Behavioral health, Workforce, Peer support, Referrals to service, Survey, Organization, Syringe services program (SSP)

## Abstract

**Background:**

Despite recent financial and policy support for harm reduction in the USA, information on the types of workers within organizations who design, implement, and actualize harm reduction services remains nascent. Little is known about how variability in the harm reduction workforce impacts referrals and linkages to other community supports. This exploratory mixed-methods study asked: (1) Who constitutes the harm reduction workforce? (2) Who provides behavioral health services within harm reduction organizations? (3) Are referral services offered and by whom? (4) Do referrals differ by type of harm reduction worker?

**Methods:**

Purposive sampling techniques were used to distribute an electronic survey to U.S.-based harm reduction organizations. Descriptive statistics were conducted. Multivariate binary logistic regression models examined the associations (a) between the odds of the referral processes at harm reduction organizations and (b) between the provision of behavioral health services and distinct types of organizational staff. Qualitative data were analyzed using a hybrid approach of inductive and thematic analysis.

**Results:**

Data from 41 states and Washington, D.C. were collected (*N* = 168; 48% response rate). Four primary types of workers were identified: community health/peer specialists (87%); medical/nursing staff (55%); behavioral health (49%); and others (34%). About 43% of organizations had a formal referral process; among these, only 32% had follow-up protocols. Qualitative findings highlighted the broad spectrum of behavioral health services offered and a broad behavioral health workforce heavily reliant on peers. Unadjusted results from multivariate models found that harm reduction organizations were more than 5 times more likely (95% CI [1.91, 13.38]) to have a formal referral process and 6 times more likely (95% CI [1.74, 21.52]) to have follow-up processes when behavioral health services were offered. Organizations were more than two times more likely (95% CI [1.09, 4.46]) to have a formal referral process and 2.36 (95% CI [1.11, 5.0]) times more likely to have follow-up processes for referrals when behavioral health providers were included.

**Conclusions:**

The composition of the harm reduction workforce is occupationally diverse. Understanding the types of services offered, as well as the workforce who provides those services, offers valuable insights into staffing and service delivery needs of frontline organizations working to reduce morbidity and mortality among those who use substances. Workforce considerations within U.S.-based harm reduction organizations are increasingly important as harm reduction services continue to expand.

## Background

Harm reduction is an evidence-based community driven approach that works directly with people who use drugs in order to prevent drug overdose and infectious disease transmission, improve the physical, mental, and social well-being of those served, and low-threshold access to substance use treatment and other health and social services [1]. Harm reduction includes pragmatic policies and programs at the individual and community levels aimed at mitigating the health and social outcomes associated with drug use [2–5]. First implemented in the Netherlands and the U.K., harm reduction was later acknowledged by the USA as a public heath response to the AIDS epidemic and harms associated with injection drug use in the 1980s, which was and remains a controversial, a primary concern being that harm reduction condones elicit behavior [2]. As an alternative to the moralistic, punitive understandings of drug use and addiction, harm reduction has been described as a *bottom-up, or grassroots approach* to engage people who use drugs (p. 779) [2]. Despite long-standing criticism toward harm reduction strategies by some U.S. policy makers, administrators, and those enforcing U.S. racialized drug-related policies [6, 7] harm reduction programs and policies have proven to be highly effective public health responses to reducing rates of infectious diseases, overdoses, morbidity, and mortality among those who use substances [1, 2, 5, 6].

### Harm reduction and community resources

Nationwide, harm reduction services have increased substantially alongside growing recognition that harm reduction provides cost-effective and evidence-based approaches to addressing individual and societal impacts of drug use [8, 9]. Harm reduction is a “*spectrum of strategies that meet people where they are―on their own terms and may serve as a pathway to …. additional prevention, treatment, and recovery services*.” [10] Because harm reduction programs do not focus on abstinence from drug use [11], they serve as a critical gateway for individuals to access important resources such as health, behavioral healthcare, and substance use treatment [8, 10, 11]. Harm reduction models reflect community-clinical linkages (CCLs), mechanisms that link individuals to services and address external barriers to care [12]. For instance, in a survey of more than 100 people who use opioids, more than half preferred a harm reduction agency as their preferred location to receive buprenorphine maintenance treatment (BMT), in contrast to traditional medical settings which they may feel unwilling or unable to access [13]. Similarly, in a randomized control trial, Strathdee and colleagues found individuals who participated in a needle exchange program (now referred to as *syringe service programs* [SSPs]) and received case management services were more likely to enter drug treatment within seven days of receiving a referral [8]. Given these examples, and in light of recent U.S. policies, state and federal agencies have made strides recognizing the important role harm reduction organizations play as a community-based resources fundamental to national efforts to address the opioid epidemic and substance use disorders [5, 12].

### Recent U.S. harm reduction policies

The U.S.’s policy response to harm reduction has varied over the decades. Despite advocacy, research, practices and policy efforts to end the “war on drugs” and promote more equitable and evidence-based drug policies [7], U.S. harm reduction policies have not aligned with expert opinion and abundant evidence [3, 6, 9]. Opposition to harm reduction is due in large part to a moralistic overtone that harm reduction encourages drug use and “NIMBYism”—a *not in my backyard* mentality that harm reduction organizations will attract undesirable people and activities into their community [14]. Recently, however, new policies have helped usher in harm reduction programing and services, including the Office of National Drug Control Policy [1].

Another important U.S. policy that has impacted harm reduction programming at the state level has been Medicaid expansion. Studies have attributed outcomes related to improved SUD/OUD treatment access and reduced overdose deaths to states who adopted Medicaid expansion compared to non-expansion states [15, 16]. Prior to COVID-19, an analysis of Medicaid expansion states revealed an 11% lower heroin death rate, and a 10% lower death rate involving synthetic opioids other than methadone, compared to states who did not expand Medicaid [16]. These findings signal how state policies may both hinder or expand harm reduction capacity and public behavioral health service systems.

At a time when more than 100,300 drug-related overdose deaths occurred in 2021—a 28.5% increase from the previous 12-month period [17]–the U.S. Department of Health and Human Services (DHHS) included harm reduction as a central pillar to its Overdose Prevention Strategy: (1) advancing research on and demonstrations of harm reduction innovation; (2) integrating harm reduction with health care delivery; (3) sustaining funding strategies for harm reduction services; and (4) reducing drug-use stigma [18]. Further, in 2021, SAMHSA announced its inaugural harm reduction grant mechanism authorized by the American Rescue Plan to invest $30 million dollars into harm reduction services and providers [5]. These new funding mechanisms are particularly important given the rate in which opioid overdose deaths have disproportionately impacted marginalized and minoritized communities [7].

Yet, despite recent financial and policy support for harm reduction, and growing literature on the role of advocacy and research evidence to advance harm reduction interventions [19–23], and implementation factors that inhibit or facilitate the uptake of harm reduction programs [24], little is known about the harm reduction workforce. How newly funded programs will be staffed, and the workforce prepared to engage in the design and implementation of harm reduction services, remains unclear. Even less is known about the behavioral health workforce involved in direct services within harm reduction community-based settings, a workforce that serves individuals with a variety of mental health and substance use needs. This exploratory study employed a mixed-methods approach to answer four research questions related to the harm reduction workforce and its ability to provide behavioral health support and community linkages. Specifically, we asked: (1) Who (i.e., which workforce types) constitutes the community-based harm reduction workforce? (2) Who provides behavioral health services at harm reduction organizations? (3) Are referral services offered and by whom? (4) Do referrals differ by the type of harm reduction worker?

## Methods

### Survey development

The survey developed for this study was created using extant literature and key informant expertise. Key informants included four individuals who worked or had prior experience working in community-based SSPs, had lived experience with substance use, and/or had experience volunteering at a SSP. During survey development, the research team sought guidance from representatives of two national organizations: The National Harm Reduction Coalition and SAMHSA. In addition, cognitive interviews were conducted with three individuals who work in harm reduction services (an executive director of an SSP, a board member for a harm reduction SSP, and a behavioral health clinician). During the cognitive interview process, clarity on response options were sought to ensure survey items were understandable and easy to navigate using the Qualtrics online survey platform.

The survey consisted of 46 questions, some open-ended and some closed-ended, designed to gather information about the community-based harm reduction workforce. The survey included four primary domains and was created based on the expert input, literature, and services found within the directory used to identify SSPs: (1) characteristics of the harm reduction organization (e.g., services provided, referral and follow-up protocols, and staff composition); (2) harm reduction strategies (e.g., behavioral health treatment, disease and overdose prevention, and formal referral services); (3) challenges related to hiring and retaining staff/workforce; and (4) respondent demographics. Questions throughout each domain offered respondents pre-populated answer choices as well a “other” category to provide an answer(s) that may have not been offered.

### Sampling techniques

Purposive sampling techniques were used to distribute an electronic survey to community-based harm reduction organizations in the USA. These organizations were identified through the North American Syringe Exchange Network (NASEN) directory of SSPs throughout the USA [21]. A master list of NASEN organizations was not shared or distributed. Rather, based on their online directory that is publicly available and periodically updated (no specific time frequency was available), contact emails for each organization were compiled by a masters’ student working with the research team to create an email distribution list. Only one person from each SSP was contacted, a limitation noted in more detail below.

### Survey delivery

The electronic survey was created using Qualtrics [25] and distributed by emailing all contacts identified with each harm reduction organization registered with NASEN. If an email address was missing, a member of the research team looked on the SSP website to find an appropriate contact. After removing duplicate emails, the survey was sent to 350 e-mail addresses in January 2023, followed by two additional reminder emails, and closed six weeks later. Qualtrics fraud protection services were used to prevent multiple survey attempts (e.g., bot detection and reCAPTCHA). The first 150 respondents were provided a $15 Amazon gift card for participation. The study was approved by the [BLINDED UNIVERSITY NAME] Institutional Review Board and electronic consent was obtained from respondents prior to starting the survey.

### Sample

The survey was intended for respondents involved in harm reduction organizations in various capacities (e.g., director, program coordinator, board member) and only those listed on the NASEN directory were contacted. The survey yielded a 48% response rate (*n* = 168) and on average took participants 10 to 15 min to complete. Most participants reported being part of executive leadership or in the director role within their organizations (76%). Respondents’ mean age was 43 years. They had an average of nine years’ experience working in harm reduction services, and an average of seven years’ experience working within their organization.

### Data analysis

Closed-ended survey results were exported from Qualtrics into Stata, which we used to conduct all quantitative data management and analysis [26]. Though our survey received 168 responses, some questions had legitimate skip patterns and Table 1, 2, 3 and 4 reflect the total number of responses for that question. Open-ended questions resulted from participants either (a) selecting “other” as a response option to a question and typing in additional information; or (b) answering a qualitative question asked directly in the survey. Qualitative data were analyzed using a hybrid approach of inductive and thematic analysis based on six steps identified by Labra and colleagues: (1) Getting familiar with the data, (2) Preliminary coding, (3) Identifying themes (round 1), (4) Assessing themes, (5) Refining and defining themes, and (6) Finalizing codes and themes [27]. Preliminary codes were generated by two team members. Next, because codes were short responses, illustrative quotes were pulled related to preliminary themes. Next, two members of the research team independently coded the responses and when this was complete, they collaboratively discussed codes and quotes. To organize the qualitative responses, Microsoft Excel was used to systematize and code open-ended responses. Finally, all coding discrepancies were resolved by the research team during weekly team meetings [27]. The codes were then organized into broader themes and integrated into quantitative findings.

**Table 1:** Respondent characteristics

Variable (total responses)	Frequency	Percentage	Mean	*SD*
Respondent age			42.82	11.42
Role (168)				
Executive Leadership	128	76.19		
Participant Services Staff	21	12.50		
Program Coordinator	19	11.31		
Respondent gender identity (147)				
Female/Woman	94	63.95		
Male/Man	37	25.17		
Transgender, genderqueer, gender non-conforming, or non-binary	15	10.20		
Prefer not to disclose	1	0.68		
Respondent ethnicity (153)				
Non-Hispanic or Latino	137	81.55		
Hispanic or Latino	16	9.52		
Respondent race (154)				
White	129	76.79		
Black or African American	10	5.95		
Other*	15	8.92		
Respondent educational attainment (152)		152		
Master’s degree or MD, or JD Doctorate/PhD,	42	25.0		
4-year degree	61	36.31		
Some college	27	16.07		
2-year associate degree/vocational degree	12	7.89		
Up to a high school degree or GED	10	5.95		
Has the respondent completed a certificate program related to harm reduction services? (153)				
No	90	53.57		
Yes	63	37.50		
Years worked in harm reduction (152)			8.86	7.86
Years respondents has worked in their harm reduction organization (149)			7.51	7.28

^*^ To ensure data are deidentified, the racial category “Other” was created to include Asian, American Indian, or Alaskan Native, and Pacific Islander/Hawaiian individuals

**Table 2:** Organization characteristics

Variable (total responses)	Frequency	Percentage	Mean (*SD*)
Syringe Services Program (SSP) has multiple sites (168)			
Yes	111	66.97	7.08 (9.80)
No	57	33.93	
Physical setting of the organization			
Primary location (168)	132	78.57	
Mobile unit (168)	86	51.19	
Pop-up sites (168)	56	33.33	
Tele-services (168)	23	13.70	
Number of unique participants served per month			350 (607.32)
Organization affiliation			
Non-profit (168)	111	66.07	
Health department (168)	53	31.55	
Faith-based (168)	11	6.55	
Other (i.e., for-profit, tribal affiliation) (168)	7	4.17	
Organization services offered		4.17	
Overdose prevention (168)^a^	163	97.02	
Disease prevention (168)^b^	154	91.67	
Community engagement (168)	163	97.02	
Testing services (168)	162	96.43	
Behavioral health services (168)	127	75.60	
Case management (127)	88	69.29	
Peer recovery (127)	78	61.41	
Counseling (127)	34	26.77	
Crisis counseling (127)	26	20.47	
Teleharm reduction (127)	23	18.11	
Types of providers/staff at organization			
Behavioral health (168)	82	48.80	
Community outreach (168)	146	87.0	
Medical (168)	92	54.76	
Other (168)	57	34.0	
Is there a formal referral process? (168)			
No	95	56.55	
Yes	73	43.45	
Is there a follow-up process for referrals? (168)			
No	114	67.86	
Yes	54	32.14	
Are there specialty mental health services? (159)			
No	80	50.31	
Yes	79	49.70	
Are volunteers essential in-service delivery (159)			
No	70	44.02	
Yes	89	55.97	

^a^Suboxone, medication assisted treatment, naloxone

^b^PEP/PREP, hepatitis A vaccine, birth control, condoms

**Table 3:** Harm reduction workforce—four primary types

Variable (each type of provider out of 168)	Frequency	Percentage
Behavioral Health Providers		
Clinical supervisors	46	27.38
Marriage and family therapists	43	25.60
Addiction counselors	38	22.62
Clinical social workers	19	11.31
Mental health/professional counselors	13	7.73
Psychiatric mental health nurse practitioners	13	7.73
Psychologists	9	5.35
Community Outreach Providers		
Community outreach specialists	114	67.86
Peer support specialists	99	58.93
Social workers	62	36.90
Advocates	32	19.05
Housing specialists	27	16.07
Insurance specialists	20	11.90
Translators	17	10.12
Case Managers	16	9.52
Job trainers	13	7.74
Promotoras	6	3.57
Medical Providers		
Physicians	40	25.0
Nurse practitioners	42	23.81
Pharmacists	16	9.52
Paramedics	8	4.76
Dentists	8	4.76
Other Types of Providers		
Grant writers	50	29.76
Researchers	14	8.33
Lawyers	12	7.14

**Table 4:** Logistic regression models assessing the odds of referral supports within harm reduction organizations

Variable	OR	95% CI	*p*-value
Formal referral process			
Behavioral health services	5.06	[1.91, 13.38]	< 0.001
Behavioral health providers	2.20	[1.09, 4.46]	0.029
Community outreach providers	1.17	[0.65, 2.14]	0.592
Medical providers	1.14	[0.90, 1.44]	0.281
Follow-up referral process			
Behavioral health services	6.11	[1.74, 21.52]	0.005
Behavioral health providers	2.36	[1.11, 5.0]	0.025
Community outreach providers	1.67	[0.86, 1.42]	0.209
Medical providers	1.10	[0.86, 1.42]	0.430

### Variables of interest

#### Independent variables

Behavioral Health Services*.* Survey respondents were asked to select all the types of behavioral health services their organization provides. From these responses, a binary variable was created to code all organizations with at least one behavioral health service offered as a 1 and all organizations without any behavioral health services as a 0.

Types of Providers in Harm Reduction Organizations*.* Survey respondents were asked to select all the types of providers who work at their organization. The types of providers were dummy coded into distinct binary variables representing four provider types: (1) behavioral health providers; (2) community outreach staff; (3) medical providers; and (4) others (e.g., grant writers and researchers). For example, organizations with at least one type of behavioral health provider (e.g., clinical social worker or licensed marriage family therapist) were coded as a 1, while organizations without any behavioral health providers were coded as a 0.

#### Dependent variables

Data for the two dependent variables in this study were obtained by asking respondents the following questions: (1) “*Does your organization have a formal referral process for participants?*” and (2) “*Do you have a follow-up process with referrals made?”* Response options were dichotomous; thus, the variables were coded as a binary. A value of 1 indicated the organization had a formal referral process and/or a follow-up process while a value of 0 indicated there was no formal referral process and/or follow-up process.

### Quantitative data analysis

Once data were downloaded and cleaned, descriptive statistics were conducted for all study variables. Next, two multivariate binary logistic regression models were conducted to examine the associations (a) between the odds of the referral processes at harm reduction organizations and (b) between the provision of behavioral health services and distinct types of organizational staff. The first multivariate model examined the association between behavioral health services, behavioral health providers, community outreach staff, and medical providers and the odds of organizations having a formal referral process. The second multivariate model examined the association between behavioral health services, behavioral health providers, community outreach staff, and medical providers and the odds of organizations having a follow-up process following a referral. Additionally, ordinal variables related to the number of participants served were created to assess if this number significantly predicted the types of behavioral health services provided at an organization, the work performed by volunteers, or the type of organization (e.g., non -profit, faith-based). However, none of these models yielded significant findings.

## Results

### Descriptive statistics

Table 1 provides demographic details about participating respondents. Organizations *(N* = *168)* represented 41 states and Washington, D.C.; organizations with the highest response rates were in California (*n* = 25) and North Carolina (*n* = 12).

Two-thirds were non-profit SSPs and about one third (32%) were SSPs operating within public health departments. Sixty seven percent of the organizations offered services across multiple sites, 51% utilized mobile units, 33% relied on pop-up sites based on community needs, and 14% offered tele-harm reduction services. On average, participating organizations reported 350 unique individuals/participants encounters per month (Table 2).

#### Harm reduction workforce composition

Among the harm reduction organizations surveyed, the included workforce composition varied with community health and peer specialists (87%; e.g., peers, outreach workers, promotoras) being the most prominent, followed by medical and nursing staff (55%; e.g., nurses, doctors), behavioral health workers (49%; e.g., clinical social workers, licensed counselors); and 34% of others which was the broadest workforce category (e.g., grant managers, legal support, administrative staff). Overall, 29% of the SSPs surveyed had all four workforce categories. Table 3 offers a breakdown of the types of providers within each category. Notably, more than half (56%) of organizations reported relying on volunteers as an essential part of harm reduction services.

Less than 50% of the SSPs had behavioral health staff and of those, 50% reported that they offered specialty mental health services—an open-ended question intended to illicit responses about more comprehensive behavioral health services offered. The types of behavioral health providers varied across organizations. The most common types of behavioral health providers included licensed marriage and family therapists (LMFTs) (26%), addiction counselors (23%), and clinical supervisors (27%); clinical social workers comprised less than 12% of the behavioral health workforce among represented organizations. However, no specific behavioral health discipline or type is discernable within the ‘addiction counselor’ and ‘clinical supervisor response options.

#### Behavioral health services

About 75% of the organizations (*n* = 127) offered any type of behavioral health service even though less than half of the organizations (49%) reported having behavioral health staff. About two-thirds of behavioral health services offered included case management (69%) and peer recovery support (61%). The behavioral health services most likely to be delivered by clinically licensed staff (e.g., clinical social workers, LMFTs) included counseling (27%) and crisis counseling (20%) (Table 2). About 43% of the organizations surveyed had a formal referral process for SSP participants; among these organizations, only 32% had follow-up protocols.

Twenty-nine percent of organizations indicated they delivered “*other types of behavioral health services.*” These responses were coded and analyzed to describe other behavioral health services delivered within these harm reduction organizations and illustrate a broad spectrum of behavioral health services offered (**Theme 1**). For example, one participant described their organization’s mental health services as “*peer-centered*” and noted that, as needed, the organization will “*contract with counselors and psychiatrists in the event that someone wants these services*.” Another said, “*our team mostly focuses on trauma care or short-term situational crisis that can be resolved in 10 sessions or less. Any longer-term behavioral health or addiction is referred to community resource.”* Although some organizations indicated they provide onsite behavioral health services, these services are often either referred off-site immediately or after a brief period of treatment.

#### Workforce providing behavioral health services

Like quantitative results in Table 2, responses to the open-ended question “*Who provides specialty mental health services in your organization*?” represented a broad behavioral health workforce engaged in harm reduction services and one that was reliant on peers (**Theme 2**). For instance, one respondent reported that their organization relied most on “*community outreach workers [who] are trained for micro counseling.*” Another stated, “*We have no employees we are totally a volunteer organization. However, we recently received opiate settlement money and we may be able to offer a paid position*.” Another respondent described the utilization of the peer workforce in the following way: “*We are a peer-run, Recovery Community Organization (RCO) that is comprised of individuals in active, long-term recovery from substance use, mental health, and trauma-related experiences. We are non-clinical and lead with our lived experience.*”

Other types of behavioral health providers identified in open-ended responses included student interns (i.e., “*we contract through a university and mental health interns*”) and volunteers.

### Association between behavioral health providers and referral patterns

In the first multivariate logistic regression model, both providing behavioral health services and having behavioral health staff within a SSP were significantly associated with the odds of harm reduction organizations having a formal referral process. The second multivariate logistic regression model also showed that both providing behavioral health services and having behavioral health staff were significantly associated with the odds of organizations having a follow-up process following a formal referral. In neither of these models was having community outreach staff or medical staff, respectively, significantly associated with the odds of an organization having formal referral process or follow-up protocols. Unadjusted results indicate that harm reduction organizations are 5.06 (95% CI [1.91, 13.38]) times more likely to have a formal referral process and 6.11 (95% CI [1.74, 21.52]) times more likely to have a follow-up referral process when they offer behavioral health services within the SSP. Additionally, SSPs are 2.20 (95% CI [1.09, 4.46]) times more likely to have a formal referral process and 2.36 (95% CI [1.11, 5.0]) times more likely to have a follow-up process for referrals when they employ behavioral health providers (Table 4).

## Discussion

The composition of the harm reduction workforce is occupationally diverse and includes many types of workers, including those with lived experiences, behavioral health providers with formal education, health professionals, and those who serve in other roles such as administrative personnel. Findings highlight the wide array of behavioral health services being offered within harm reduction organizations (**Theme 1**) and the broad composition of the behavioral health workforce involved in harm reduction services within U.S.-based community SSPs, with reliance on the peer workforce (**Theme 2**). Further, we found that having behavioral health workers as part of the harm reduction team facilitated organizations’ formal referral and follow-up processes, an important aspect of harm reduction and community linkages. Assessing the referral and follow-up protocols of SSPs allows organizations to identify if those community connections occur and by whom.

Harm reduction has been led by diverse community groups to advance social justice and to more aptly meet the needs of diverse populations (e.g., LGBTQ, sex workers) [4]. Central to the U.S.-based National Harm Reduction Coalition is to encourage individuals and organizations “to be a catalyst for love, justice, community and connection.” [4] As Evidence has shown that SSPs can provide a bridge to various forms of service delivery [2, 28] and operate as a “*low-threshold gateway to welcome anyone who is willing to ‘come as they are’*” (p. 788) [2]. Findings from this study not only confirm that this bridging to additional services occurs; they also indicate that the composition of the harm reduction workforce can enhance how these referrals and follow-up protocols are actualized. Specifically, having behavioral health workers within SSPs facilitates significantly more referrals to additional community, social, and health supports compared to other groups of workers identified (e.g., medical staff and community outreach workers). However, our analyses showed that the behavioral health workforce is occupationally diverse and includes those with lived-life experience, varied levels of education, and professional training.

While making behavioral health as a diverse service category may increase the number of people working to address behavioral health needs, it may also produce greater variation in what services are offered and by whom. Variation in scope of practice, training, and skills vary by workforce type along with payment mechanisms that reimburse or financially support different types of work performed [29]. As such, investigating the variation and scope of practice within the behavioral health workforce, as well as how this workforce is reimbursed and paid for harm reduction services, is necessary to determine how to scale behavioral health supports for SSP participants to improve service delivery and other supports as need. Future research should also assess whether the type(s) of behavioral health providers in a SSP result in different referral and follow-up processes and how this impacts SSP participant outcomes.

Notably, our findings highlight the organization’s adherence to a central premise of harm reduction philosophy and practices: “*meeting people where they are*.” [30, 31] Community outreach specialists were the most common type of workforce identified within this study (87%), and peer recovery services were the most offered behavioral health service. Given that peer recovery services comprised almost two-thirds of all behavioral health services offered, future research should unpack who these peers are, what training they have, and how they are prepared to work in the harm reduction field given the severity of use and life-threatening consequences related to substance use. Prior research has documented the benefits of leveraging the peer workforce to address behavioral health needs and the recent nationwide proliferation of the peer behavioral health workforce [32, 33], especially in light of the behavioral health challenges and social vulnerabilities associated with COVID-19 [34, 35] and increasing rates of overdose deaths [17]. Recently, the Biden-Harris Administration identified peer supports as essential to accelerating the mental health workforce and improving health care delivery [36, 37]. However, understanding how the harm reduction field and SSP organizations attract, train, and retain peers is a critical yet unexplored aspect of harm reduction workforce projections and planning. Although prior research has given attention to reimbursement for peer-led services, supervision, and required and desired training to support the peer workforce [32, 38], these models have not focused on harm reduction specifically. Determining how best to strengthen the harm reduction workforce, and the peer support specialist workforce in particular, is essential to effectively delivering harm reduction services. Current efforts to incorporate peers as part of the workforce to address the U.S.’s behavioral health crisis will continue to be important especially as harm reduction organizations continue to rely on their expertise as integral members of the harm reduction workforce.

Increasing the behavioral health workforce within the field of harm reduction could also increase the provision of behavioral health treatment (i.e., SBIRT, motivational interviewing, psychotherapy), to people in their own community, rather than requiring them to attend mental health treatment in a different locale/setting. The relatively smaller percentages of licensed behavioral health providers (e.g., psychologists and clinical social workers) we observed may stem from reimbursement mechanisms and policy restrictions on what types of behavioral health providers can bill for clinical behavioral health services [39]. While this survey did not explore the funding models of each program, it is plausible that these types of behavioral health providers are less prevalent in these settings because behavioral health service reimbursement (i.e., insurance access and billing) is not a common funding mechanism used to sustain harm reduction and SSP community-based programming [40]. As harm reduction services and behavioral health supports become more commonly understood, and more and more states expand Medicaid, funding and reimbursement may emerge as an increasingly important source of revenue for SSPs. Recent work from the Center for Health Care Strategies in partnership with The Pew Charitable Trusts has developed guiding principles for states to expand harm reduction services through publicly funding financing mechanisms to increase access to substance use care, treatment, and support services [40]:

Opportunities to build infrastructure have been scarce and few harm reduction provider organizations are approved Medicaid providers with adequate billing capacity. States can consider providing support to community-based harm reduction providers to develop the infrastructure necessary to bill Medicaid for allowable services, including providing technical assistance (TA) and guidance to programs that may have concerns regarding patient data collection requirements. States can assess opportunities to relax data collection requirements where possible and work closely with harm reduction partners to develop data collection processes that are informed by people who utilize these services. [40]

As states expand harm reduction services, more research is needed to further understand what policy drivers may be impacting the types of behavioral health workforce hired by SSPs.

### Strengths and limitations

To the best of our knowledge, this is the first national survey assessing behavioral health issues among harm reduction organizations in the U.S. Findings from this study may have significant implications for future funding and policy changes – specifically, for increased funding for staff positions and the expansion of behavioral health services in harm reduction organizations. However, our findings should be interpreted considering study limitations. First, while our survey comes from a national sample of registered SSPs within the NASEN network and achieved a 48% response rate, there is limited generalizability given organizations not part of the NASEN directory were not included in this sampling frame. Second, the cross-sectional nature offers a single snapshot in time and may not reflect a static national profile of harm reduction organizations, nor can causality be determined [41]. Additionally, only one person from each SSP was contacted and thus, different perspectives were not sought per each SSP. Fourth, among the SSPs that responded, there is considerable variation in organizations’ size and geographic location. This means that, for example, unique considerations for SSPs in urban vs. rural areas of the U.S. or based on state policies that may inhibit or enhance harm reduction services may have potentially skewed our findings. Further, when looking at types of providers and services, the specific types of behavioral health provider (i.e., addiction counselor) was not always distinguishable and could include someone with a social work degree, a peer support specialist, among others. The authors also acknowledge that the analysis is exploratory and does not consider state-specific policies (e.g., Medicaid expansion, laws around criminalization of drug use, sex work, and other mechanism of marginalization) or geographic variability which will be assessed in future work. Finally, researchers assessed if organizations had a referral process, without noting which services were referred to and the outcome of these referrals.

Notwithstanding these limitations, this study calls attention to the essential and lifesaving services provided by harm reduction organizations across the USA. This study also calls for future research to examine the challenges and barriers to hiring and sustaining the harm reduction workforce, and the ways in which organizations rely on peers and volunteers to carry forward harm reduction services with vulnerable populations. Future research and evaluative efforts could assess sustainable streams of funding for harm reduction organizations to ensure there is adequate staff to deliver a variety of services (e.g., SSPs, behavioral health, and medical care).

## Conclusions

The workforce diversity of harm reduction organizations highlights how interdisciplinary teams are working together to engage individuals seeking harm reduction services. The behavioral health workforce within harm reduction varies by provider types (i.e., lived-life, professional, and educational experiences) and by the type of behavioral health services offered. Nonetheless, including behavioral health providers within SSPs significantly increases SSPs referrals and follow-up protocols to extend the harm reduction continuum of care. Given that harm reduction organizations serve individuals with a multitude of needs including physical health concerns, behavioral health, crisis situations, social and basic needs, further analysis of the harm reduction workforce can help ensure that comprehensive and coordinated services are delivered to participants accessing SSPs. Understanding the types of services offered, as well as the workforce provides those services, offers organizations valuable insights into staffing and service delivery strengths, and needs by frontline organizations working to reduce morbidity and mortality among those who use substances.

## Data Availability

The survey and data collected were done so by the research team and are not publicly available.

## Abbreviations

CDC: Centers for Disease Control and Prevention
CI: Confidence interval
DHHS: U.S. Department of Health and Human Services
LMFT: Licensed marriage and family therapist
NASEN: North American Syringe Exchange Network
RCO: Recovery community organization
SAMHSA: Substance Abuse and Mental Health Services Administration
SSP: Syringe service program
